# A Novel Combined Dry Powder Inhaler Comprising Nanosized Ketoprofen-Embedded Mannitol-Coated Microparticles for Pulmonary Inflammations: Development, In Vitro–In Silico Characterization, and Cell Line Evaluation

**DOI:** 10.3390/ph17010075

**Published:** 2024-01-07

**Authors:** Heba Banat, Ildikó Csóka, Dóra Paróczai, Katalin Burian, Árpád Farkas, Rita Ambrus

**Affiliations:** 1 Institute of Pharmaceutical Technology and Regulatory Affairs, Faculty of Pharmacy, University of Szeged, Eötvös u.6, 6720 Szeged, Hungary; banat.habosh@gmail.com (H.B.); csoka.ildiko@szte.hu (I.C.); 2 Department of Medical Microbiology, Faculty of Medicine, University of Szeged, Dóm Square 10, 6720 Szeged, Hungary; paroczai.dora@med.u-szeged.hu (D.P.); burian.katalin@med.u-szeged.hu (K.B.); 3 Centre for Energy Research, Hungarian Academy of Sciences, 1121 Budapest, Hungary; farkas.arpad@ek-cer.hu

**Keywords:** pulmonary delivery, mannitol, ketoprofen, milling, spray-drying, particle engineering, combination product, inflammation, nano-in-micro

## Abstract

Pulmonary inflammations such as chronic obstructive pulmonary disease and cystic fibrosis are widespread and can be fatal, especially when they are characterized by abnormal mucus accumulation. Inhaled corticosteroids are commonly used for lung inflammations despite their considerable side effects. By utilizing particle engineering techniques, a combined dry powder inhaler (DPI) comprising nanosized ketoprofen-embedded mannitol-coated microparticles was developed. A nanoembedded microparticle system means a novel advance in pulmonary delivery by enhancing local pulmonary deposition while avoiding clearance mechanisms. Ketoprofen, a poorly water-soluble anti-inflammatory drug, was dispersed in the stabilizer solution and then homogenized by ultraturrax. Following this, a ketoprofen-containing nanosuspension was produced by wet-media milling. Furthermore, co-spray drying was conducted with L-leucine (dispersity enhancer) and mannitol (coating and mucuactive agent). Particle size, morphology, dissolution, permeation, viscosity, in vitro and in silico deposition, cytotoxicity, and anti-inflammatory effect were investigated. The particle size of the ketoprofen-containing nanosuspension was ~230 nm. SEM images of the spray-dried powder displayed wrinkled, coated, and nearly spherical particles with a final size of ~2 µm (nano-in-micro), which is optimal for pulmonary delivery. The mannitol-containing samples decreased the viscosity of 10% mucin solution. The results of the mass median aerodynamic diameter (2.4–4.5 µm), fine particle fraction (56–71%), permeation (five-fold enhancement), and dissolution (80% release in 5 min) confirmed that the system is ideal for local inhalation. All samples showed a significant anti-inflammatory effect and decreased IL-6 on the LPS-treated U937 cell line with low cytotoxicity. Hence, developing an innovative combined DPI comprising ketoprofen and mannitol by employing a nano-in-micro approach is a potential treatment for lung inflammations.

## 1. Introduction

Inflammations are the body’s normal reaction to both infectious and non-infectious injuries, and it triggers a wide range of intricate systems that ultimately result in tissue repair. Infections, as well as exposure to chemicals, allergens, and irritants, are the most frequent causes of an inflammatory response in the lungs [1]. Normal inflammation is meant to be protective, but when it is extensive or lasts for long time, it can have negative effects that lead to bad consequences [2]. Numerous inflammatory cell subtypes are activated during lung inflammations; each one releases mediators and proinflammatory cytokines (i.e., IL-6, IL-8, and TNF-α) to control the actions of other inflammatory cells, which leads to a serious inflammatory progression [3,4]. Diseases such as chronic obstructive pulmonary disease (COPD), cystic fibrosis (CF), bronchiectasis, and bronchitis are manifestations of chronic pulmonary inflammation [5,6,7]. Pulmonary inflammations are featured by hypersecretion of mucus throughout the airways and lungs [8]. In the physiological and pathological processes that occur in the airways, mucus plays a significant role. Besides its function in mucociliary clearance by entrapping and removing bacteria and other inhaled irritants, it also protects, hydrates, and softens mucosal surfaces [9]. However, mucus overproduction impairs mucociliary clearance and causes obstructive airways [10]. Excessive inflammations can be fatal, and therefore, finding new treatment approaches is an imperative need.

Inhaled products have shown their effectiveness by targeting lung diseases locally while avoiding systemic exposure, with fewer adverse effects. Inhaled corticosteroids (ICSs) are commonly used in lung inflammations (i.e., COPD). Nevertheless, administration of ICSs over a long time shows serious side effects, not only locally (i.e., oral candidiasis, dysphonia, hoarse voice, and cough) [11], but also systemically (i.e., cataracts, weakening and bruising of the skin, and impaired bone mineral density) [12]. It was proven that lung inflammation was suppressed by the cyclogenase-2 (COX-2) inhibitor, which may help in the recovery of the bronchial epithelium structure [13]; therefore, inhaled non-steroidal anti-inflammatory agents (NSAIDs) could be a beneficial alternative to ICSs. Ketoprofen (KETO) is a well-known NSAID that acts as an inhibitor of COX-2 and cyclogenase-1 (COX-1) [14,15]. KETO is available on the market for oral, topical, and rectal administration but not for pulmonary administration (https://go.drugbank.com/drugs/DB01009, accessed on 28 May 2023). Nevertheless, a few studies have been conducted to develop KETO for inhalation in its salt form (ketoprofen lysinate) by a single step of co-spray drying with leucine [16,17]. D-Mannitol (MAN) is categorized as a sugar alcohol that can work as an osmotic agent [18]. Inhaled MAN is commercially available (Bronchitol^TM^) in Europe as a dry powder inhaler (DPI) and used as an adjuvant maintenance treatment with a dosage of 400 mg twice daily for patients with CF. When MAN is inhaled, an osmotic gradient is created that allows for the outflow of water into the airway lumen, in which the water content is increased at the surface of the airway and mucus clearance is facilitated through ciliary activity and coughing [19].

Particle engineering is an adaptable technique and considered an essential factor for the development of DPIs, the most common form of pulmonary delivery, in which the surface of drugs, carriers, or excipients can be controlled [20]. To balance the interparticle forces between drug particles, maintain appropriate stability during processing and storage, and improve aerodynamic properties, particle engineering can be employed in the formulation of combined DPIs [21]. Among particle engineering techniques, a spray dryer can create powder for inhalation with optimum characteristics. Due to its ability to modify parameters including temperature, flow rate, liquid feedstock concentration, and mesh size, a spray dryer has a variety of features that precisely facilitate dried powder formulations [22,23]. Liquid mixtures (solutions, suspensions, or emulsions) that contain drugs are transformed into a dry powder using the single-step manufacturing process of spray-drying. When drugs are water-soluble, spray-drying is preferable, since the dry powder for inhalation can be formed directly from an aqueous medium [24]. However, for poorly water-soluble drugs, further steps should be employed. Media milling is a “top-down” technique that can be applied to poorly water-soluble drugs in order to reduce their particle size to the micro or nano range, thus modifying their physico-chemical characteristics. Media milling has been employed to develop inhaled products containing poorly water-soluble drugs [25].

Particle size is the primary concern for pulmonary delivery. While the optimum particle size for lung deposition is less than 5 microns, nanoparticles are needed for targeting the drug to deep lung regions with enhanced dissolution, permeation, and aerodynamic profile [26,27,28,29]. However, inhalation of nanoparticles can either be exhaled or removed by lung defense mechanisms (i.e., mucociliary clearance). Therefore, microsized particles (~2 µm) are preferable in order to ensure the required deposition. Nano-in-micro is a promising approach that has been developed for DPIs to embed the drug with nanosize (<0.5 µm) in microparticles (<5 µm), taking advantage of optimized lung deposition and bypassing clearance mechanisms [30,31].

In this study, we aimed to develop a novel combined DPI comprising KETO and MAN. To the best of our knowledge, this is the first time the combination of KETO and MAN for pulmonary delivery haas been studied by employing a “nanoparticle-embedded coated-microparticle” system. In order to check the compatibility of this combination, the influence of MAN on the habit of KETO was studied. Analyses of solubility, drug content, particle size, morphology, dissolution, permeation, structural and thermal study, in vitro and in silico deposition, mucin viscosity, cytotoxicity, and the anti-inflammatory effect were conducted. Hence, our “nano-embedded coated-microparticles” system can be considered an innovative model of combination products in a single DPI for targeting pulmonary inflammations (using KETO nanoparticles) and simultaneously improving the mucus clearance (using the MAN layer).

## 2. Results

### 2.1. Holding Time as Short-Term Stability of KN

**Figure 1:**
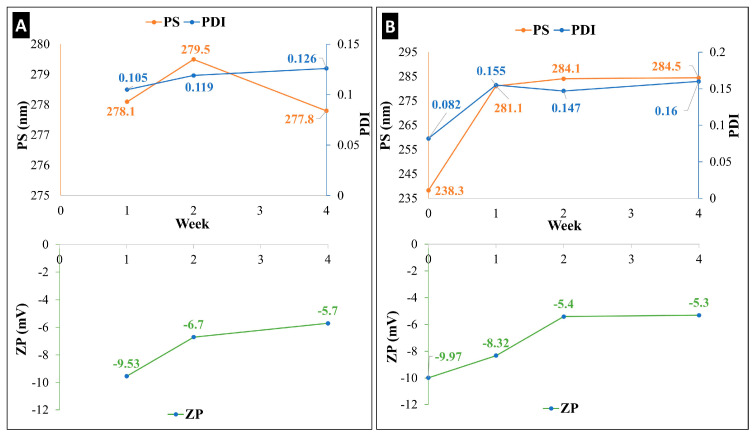
Holding time as short-term stability (four weeks) of ketoprofen-containing nanosuspension (KN), characterized by PS, PDI, and ZP at two temperatures: (**A**) +4 °C and (**B**) 25 °C.

By assessing particle size (PS), polydispersity index (PDI), and zeta potential (ZP) values at 25 °C and +4 °C for one month, the produced ketoprofen-containing nanosuspension (KN) underwent a short-term physical stability investigation (Figure 1). The KN showed a higher stability when stored in the refrigerator (Figure 1A) than at room temperature (Figure 1B). After 4 weeks, no significant difference was observed in terms of PS and PDI when the KN was kept refrigerated (+4 °C), while at room temperature (25 °C), a growth of 50 nm in PS and a double growth in PDI were found. A lower ZP value was recorded at both temperatures; however, it is still in line with other formulations that are prepared with Poly-vinyl-alcohol (PVA) as a stabilizer. It is necessary to highlight here that the use of sodium dodecyl sulphate (SDS) as an anionic surfactant played a crucial role in enhancing the stability of the KN by maintaining the electrostatic stability [32]. Many studies showed that a more stable nanosuspension can be gained when the stabilizers/surfactants are applied in combination [33]. After passing this stability test, the nanosuspension was qualified to enter the solidification process and be further processed into a dry powder.

### 2.2. Yield and Drug Content of DPI

Table 1 represents the sample notations, description of samples, percentage yield, and drug content. Samples were named according to the mass ratio of MAN and KETO as explained in Section 4.3. The spray-dried samples showed a percentage yield of 53–59%. Increasing the concentration of MAN had a positive influence on the yield, since it reduces the cohesive forces between particles. The presence of cohesive particles is indicated by low spray-drying yields [34]. Our results are comparably higher than other studies using the spray-drying technique [35,36,37]. Similarly, the drug loading results recorded between 57% and 85%. However, there was no substantial effect for MAN concentration (F0.5–F2).

### 2.3. Particle Size, Particle Size Distribution, and Zeta Potential

The outcomes displayed in Table 2 demonstrated that the particle engineering strategies that we employed were successful in reducing the PS of KETO. It was found that there was no significant rise in the PS of KETO after the spray-drying process, which implied proper redispersible formulations with no aggregation. Our samples showed a better ZP result compared to prior studies, in which PVA was used as a stabilizer for pulmonary delivery [38]. The highest PDI was found in F0 (no MAN), which indicated a better dispersity enhancement in the case of samples with MAN.

### 2.4. Solubility

The solubility study was carried out for KETO and our spray-dried formulations (F0, F0.5, F1, and F2) in distilled water at room temperature with continuous stirring for 24 h. Ketoprofen is assigned to BCS Class II, with low solubility. As shown in Table 3, KETO recorded a solubility of 0.42 mg/mL. The maximum solubility was 17.95 mg/mL in the sample with the highest concentration of MAN (F2). The spray-dried samples showed a 42.5-fold increase in the solubility compared to KETO. The explanation for the improvement in solubility can be attributed to the increase in surface area caused by the nanosize of the KETO. This can predict a better dissolution and better diffusion profiles. Thus, the solubility results confirmed that the preparation techniques were successful and can be considered for the formulation of poorly water-soluble compounds.

### 2.5. Morphology

**Figure 2:**
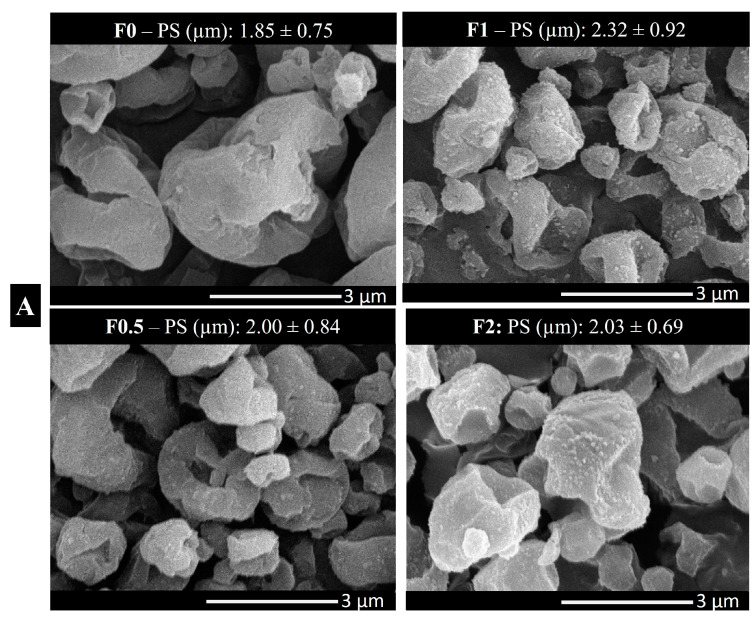

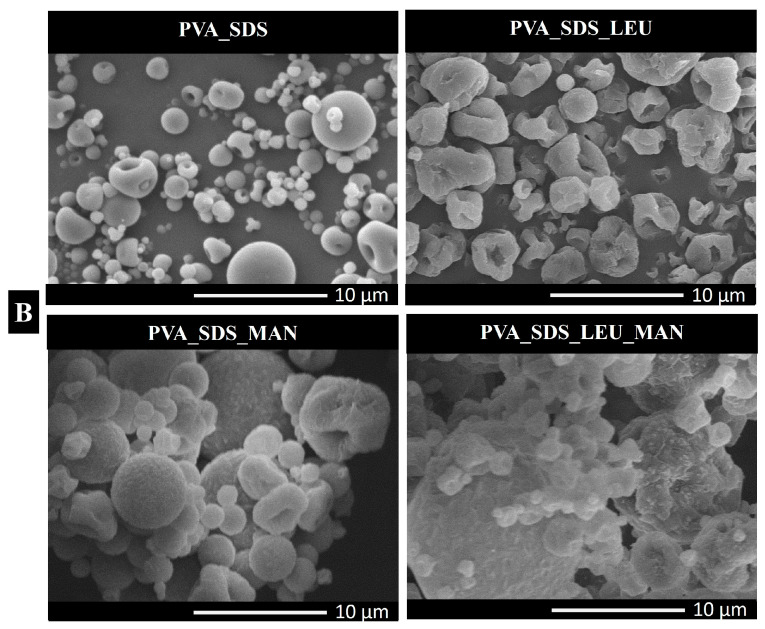
Morphology images using scanning electron microscope (SEM): (**A**) spray-dried samples and the diameter of final product measured by Image-J software; (**B**) spray-dried blanks (PVA_SDS, PVA_SDS_LEU, PVA_SDS_MAN, and PVA_SDS_LEU_MAN). PS: particle size. Data are mean ± SD (n = 3 independent measurements).

Figure 2A illustrates SEM images of spray-dried samples (F0–F2). All particles revealed a nearly spherical shape with a rough surface. The spherical form of particles for pulmonary delivery is the most stable form [39]. However, the rough surface is preferable for lung deposition, since it increases the contact angles and thus enhances the attachment on the lung cell due to a large surface area [40,41]. Image-J software was used to measure the PS of our final formulations. Images were taken in different magnifications, and 50–100 particles were selected for the size measurement. The particle size of the final products proved the nano-in-micro approach. Figure 2B illustrates the spray-dried blanks (PVA_SDS, PVA_SDS_LEU, PVA_SDS_MAN, and PVA_SDS_LEU_MAN). The SEM images of blanks were needed to confirm the behavior of LEU and MAN in combination after spray-drying. MAN, being more soluble, tends to crystallize and deposit on the surface of the particles, acting as a coating material [42].

### 2.6. Contact Angle, Surface Energy, and Cohesion Work

**Figure 3:**
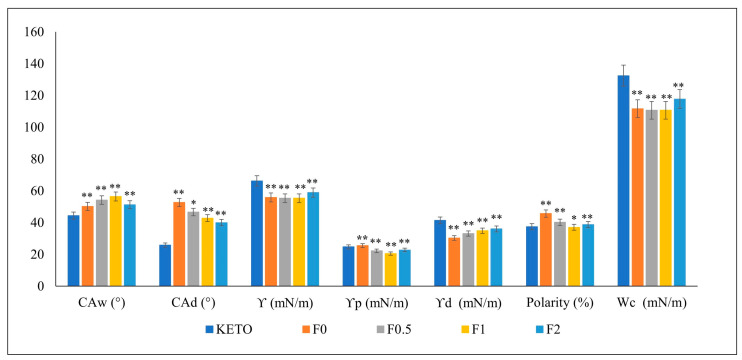
Contact angle, surface energy, polarity, and cohesion work. CAw: contact angle in water, CAd: contact angle in diiodomethane, Υ: surface energy, Υp: polar part, Υ: dispersive part, Wc: cohesion work. Results are expressed as mean ± SD (n = 3 independent measurements). Level of significance compared to KETO (* *p* < 0.05), (** *p* < 0.01).

The interparticle interactions of the spray-dried samples and KETO were investigated using the OCA apparatus (Figure 3). The surface energy is a measurement of the cohesive forces within the particles and their interaction with the surroundings. The higher the surface energy is, the higher the cohesive force which leads to low wettability is. The dissolution of solid drugs requires particle wetting, which is principally controlled by powder surface energetics [43]. All the formulations showed lower surface energies compared with KETO. Moreover, samples F0.5–F2 showed a decreased polarity compared to F0 because of MAN, which acts as a wetting agent. This predicted an enhancement in both dissolution and diffusion. The statistical analysis of the data was performed with GraphPad Prism 8.0.1. software, using one-way ANOVA. A *p* value < 0.05 was considered statistically significant.

### 2.7. Thermal Analysis

#### 2.7.1. DSC

**Figure 4:**
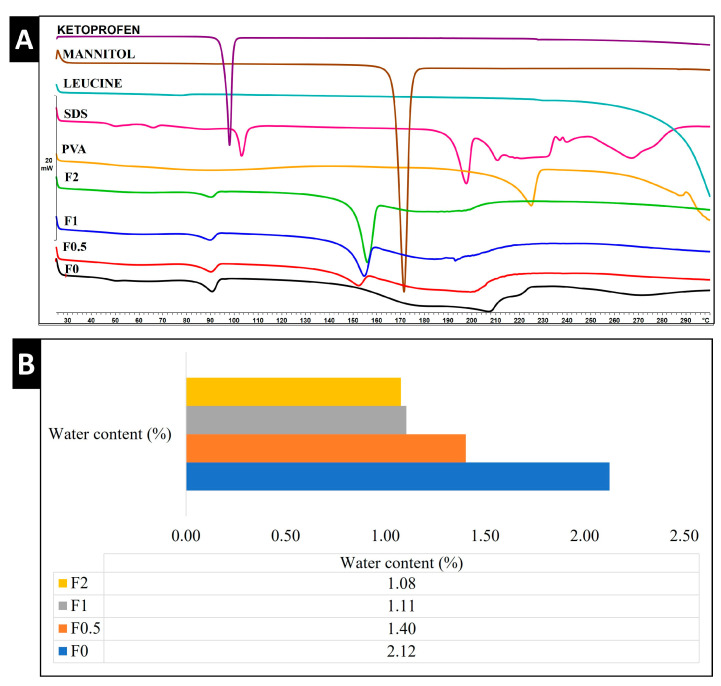

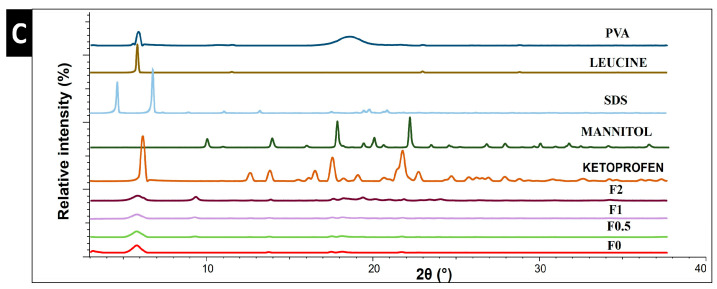
Thermal and structural analysis of raw materials, spray-dried samples, and physical mixture. (**A**) DSC, (**B**) water content by TGA, and (**C**) XRPD. PM: physical mixture.

DSC thermograms of the raw ingredients (KETO, LEU, MAN, PVA, SDS), physical mixtures (PM0–PM2), and samples (F0–F2) were obtained to assess the thermal behavior of the nanoparticles within our formulation (Figure 4A). One endothermic peak at 95.32 °C was demonstrated in raw KETO, which is equivalent to its melting point. Meanwhile, MAN expressed its melting point by a very sharp endothermic peak at 171.33 °C. Those sharp peaks are an indication of a crystalline form. SDS and PVA showed endothermic peaks at 103.00 °C and 224.83 °C, respectively, whereas no endothermic nor exothermic peaks were detected for LEU, and its degradation appeared at 294.41 °C. Two broad endothermic peaks were found in F0.5, F1, and F2, while a single broad endothermic peak was recorded in F0 (no MAN). This indicated that a partial crystallinity of KETO existed. The melting points of KETO in samples F0–F2 were recorded at a lower temperature (~90 °C) compared to raw KETO due to its presence in nanosize and the increase in amorphous structures. However, it was noted that the peaks of KETO became smaller and broader by increasing the ratio of MAN. It was confirmed that MAN has a decreasing effect on drug crystallinity when used as an excipient [44]. Those results were correlated to the XRPD. The DSC results showed that the outer coating layers of MAN were partially crystalline in samples F0.5, F1, and F2, with peaks recorded at a lower temperature (152.17 °C, 154.50 °C, and 155.83 °C, respectively). The presence of MAN in crystalline form enhanced the stability and improved both in vitro dissolution and diffusion profiles in pulmonary delivery [45,46].

#### 2.7.2. TGA

DPI formulations must have a low water content in order to efficiently aerosolize, disperse, and reach the lungs. Our samples detected a residual water content that ranged from 1.08% to 2.12%, as shown in Figure 4B. The water content of spray-dried powder is reported in the literature to exhibit ranges of 0.24%, 4.1%, and 9.02% [47,48]. Hence, our formulation can be deemed to have a lower water content in comparison with previous studies.

### 2.8. Structural Analysis

In this study, XRPD was employed to characterize the crystallinity state. Figure 4C illustrates the diffractograms of raw materials (PVA, LEU, SDS, MAN, and KETO) and samples (F0–F2). Raw KETO showed 2-theta (2θ) peaks at 5.79°, 12.9°, 14.20°, 17.12°, 18.30°, and 21.83°, which correspond to its intense crystallinity. Characteristic peaks of raw MAN were found at 14.2°, 10.4°, 18.40°, 20.30, and 22.2° 2θ, indicating its crystalline state. All formulations showed characteristic peaks with low intensity, demonstrating that they partially converted into an amorphous state during the preparation process. To obtain more accurate data about the crystallinity phenomena in samples F0–F2, the areas beneath the curves were calculated and compared with raw KETO. The crystallinity percentages of KETO were 60.30%, 44.54%, 41.96%, and 28.36% for samples F0, F0.5, F1, and F2, respectively. These results demonstrated that MAN lowered the crystallinity, which is in line with the literature [44].

### 2.9. Density and Flowability

Table 4 demonstrates the density characteristics. Our formulations illustrated a low tapped density of 0.18–0.22 g/cm^3^, which is considered optimal for pulmonary delivery. The value of 0.3 g/cm^3^ can be regarded as a limit, because previous results showed that above this point, it is difficult to accomplish appropriate aerodynamic results [49,50]. In the DPI formulation, a deeper airway’s flowability and deposition may be improved by the decreased density. Also, a higher respirable fraction can be obtained by a lower tapped density [51]. The HR and CI results are correlated with the aerosol performance. The results of HR and CI were in line with other carrier-free DPI formulations [52].

### 2.10. Aerosol Performance

#### 2.10.1. In Vitro Aerodynamic Characterization

**Figure 5:**
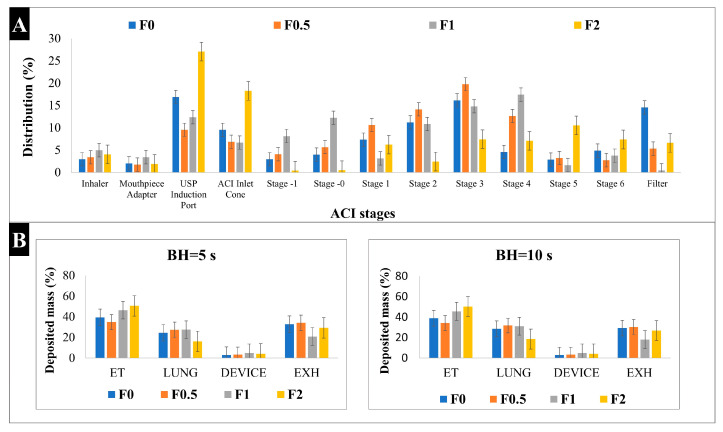
Aerosol performance characterization: (**A**) In vitro distribution of spray-dried samples by Andersen Cascade Impactor (ACI); (**B**) in silico results of deposited mass of spray-dried samples. BH: breath holding time, ET: extra-thoracic, EXH: exhaled. Results are expressed as mean ± SD (n = 3 independent measurements).

The Andersen Cascade Impactor (ACI) stages are created in a manner so that they can mimic the pattern of pulmonary deposition. The aerodynamic characteristics that were analyzed by the ACI at a 60 L/min flow rate exposed that our samples are suitable for lung deposition. Table 4 shows the results for the mass median aerodynamic diameter (MMAD), fine particle fraction (FPF), and emitted fraction (EF). The MMAD for the spray-dried samples was between 2.4 μm and 4.9 μm, which is optimal for lung deposition. However, it was noticeable that the higher the MAN ratio was, the higher the MMAD was. On the other hand, MAN enhanced the FPF from 56.16% to 71.02% and 64.32% in F0.5 and F1, respectively. These FPF recordings are higher than other products that are available on the market [53], which can be considered promising for deeper lung deposition. All samples had an acceptable EF result between 95% and 97%, putting them in line with the aerodynamic particle size distribution (APSD) testing requirement, in which the value must be between 85% and 115% [35]. Figure 5A illustrates the deposition distribution (%) in the ACI stages. While F0 and F2 are highly deposited in the USP induction port (17% and 27%, respectively), F0.5 and F1 depositions are mainly in stages 3 (20% and 15%, respectively) and 4 (13% and 18%, respectively), which represent the deep region in the lungs. This confirmed that the addition of MAN in concentrations either half or equal to KETO enhances the lung deposition.

#### 2.10.2. In Silico Characterization

For pulmonary delivery, the in silico model results mostly concurred with in vivo studies [54]. In our study, the in silico model was applied for a better understanding of the aerosol’s performance of our samples in the airways. Based on the results shown in Figure 5B, all samples led to a higher lung deposition when the breath holding (BH) time was increased to 10 s. For example, lung deposition at 5 s was 24.6%, 27.5%, 27.6%, and 16.2%, while at 10 s, it was 28.7%, 31.8%, 31.2%, and 18.6% for samples F0, F0.5, F1, and F2, respectively. Also, all samples showed a lower exhaled amount at 10 s compared to 5 s.

The results obtained here were in accordance with the patterns identified by the in vitro measurements. However, the in silico models can show lower lung deposition values compared to the in vitro methods (i.e., ACI) [55]. The high extra-thoracic (ET), upper airways, and deposition results here were due to the shorter inhalation time used (2.04 s), which is half the inhalation time that was used in ACI. It was proven that the amount of drug particles deposited in the deep lung shows a two-fold increase when the inhalation time is increased [56,57]. Hence, the highest lung deposition fractions were found in F0.5 and F1, which is correlated with in vitro results. These results are somewhat optimistic, since the in silico model better simulates the actual lung deposition in real time, and this supports our aim of targeting pulmonary inflammations.

### 2.11. In Vitro Release Study

**Figure 6:**
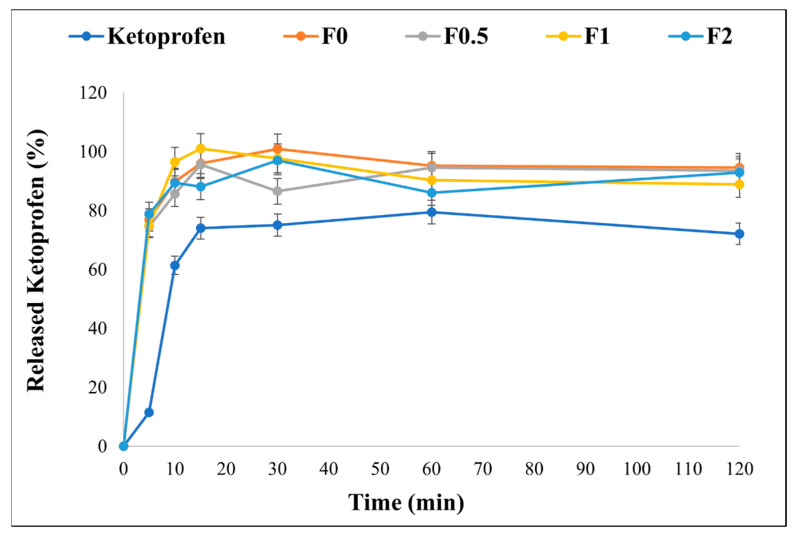
In vitro release profile of raw KETO and spray-dried samples in simulated lung media, pH = 7.4. Results are expressed as mean ± SD (n = 3 independent measurements).

The in vitro dissolution test was conducted for 2 h in a simulated lung media (pH 7.4) to mimic lung conditions. As mentioned previously, KETO is a poorly water-soluble drug, and its release profile is shown in Figure 6. It is known in pulmonary delivery that the delivered medication dose must be quickly dissolved and released upon lung deposition to reduce the risk of a clearance mechanism (i.e., macrophage uptake) [38,58]. It is demonstrated that ~80% of the drug released from our formulations within the first 5 min, while only 11% released from the raw KETO. The enhancement in release profile is due to the nanosized KETO particle and the existence of MAN. While all spray-dried samples showed an enhanced release profile, the fastest dissolution was recorded by F1, where 100% of the drug released within the first 10 min. Since MAN is highly wettable, it can speed up the disintegration and help the drug release [59]. However, the addition of MAN in a higher concentration might restrict a more complete liberation of the drug. Also, in F0.5, the MAN concentration was not sufficient to signifyingly enhance the dissolution of KETO. Therefore, the selection of a suitable concentration should be evaluated for other formulations.

### 2.12. In Vitro Diffusion Study

Since the lungs enable drug delivery in a very low dose (1/10 of oral dose), this dose needs to be diffused in the lung cells to give the required local action [60]. The permeation of our formulations and raw KETO was assessed from a simulated lung fluid to the epithelium through a cellulose membrane (soaked in isopropyl myristate) to simulate the real conditions of the lungs. Table 5 demonstrates the results of the permeated amount (µg/cm^2^) of raw KETO and spray-dried samples. All samples showed a better diffusion compared to the raw KETO; this is mainly due to the nanosized drug particle. The highest permeated amount (~122 µg/cm^2^) after 60 min was detected in F1, which is equal to a five-fold enhancement compared to raw KETO (24.8 µg/cm^2^). Due to the increased surface area and the hyperosmotic effect created by the nanoembedded coated microparticle system, a significant amount of KETO could diffuse to epithelium from samples. These findings were associated with the in vitro dissolution test. Consequently, this combined system may be beneficial for the local management of pulmonary inflammation.

### 2.13. The Effect on Mucin Viscosity

Abnormalities of mucus viscosity play a critical role in the pathogenesis of several respiratory diseases [61]. Therefore, we studied the effect of our formulations on the viscosity of mucin (the major component of mucus). As a preliminary study, the mucin solution was prepared in three different concentrations: 2%, 5%, and 10%. Those concentrations were chosen based on previously reported studies. The total mucin concentration of 2% was reported by [62,63], while 10% was reported by [64], and 5% was chosen as a middle point. In our study, the 2% mucin-containing solution was so diluted that its viscosity was unable to be detected, while 5% and 10% showed a viscosity of 0.019 and 0.035 Pa·s, respectively. The viscosity of mucus was reported in cystic fibrosis and COPD patients as 0.03–0.38 Pa·s and 0.04–1.8 Pa·s, respectively [65,66]. Therefore, a 10% concentration of mucin was chosen for investigating the effect of our samples. F1 showed the lowest viscosity, while none of the samples (F0, F0.5, and F2) decreased the viscosity significantly (Table 6). Lowering the mucin viscosity can be associated with an increase in hydration (due to MAN), and reducing the viscosity of mucin can contribute to improved mucus clearance. Further investigations should be carried out to evaluate the correlation between MAN concentrations in our samples and the viscosity of sputum collected from COPD and CF patients.

### 2.14. Cytotoxicity Study

**Figure 7:**
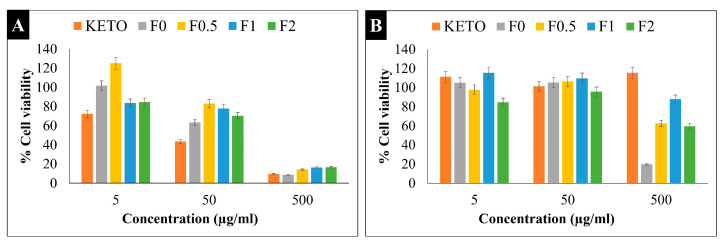
MTT viability study. Percentage of cell viability at different concentrations on (**A**) U937 and (**B**) A549 cells. Results are expressed as mean ± SD (n = 4 independent measurements).

An MTT viability study was carried out on two types of cell lines (A549 and U937). The A549 cell line is an extensively used cell model for inhaled products which represents the human alveolar epithelium. These cells have an epithelial type II phenotype, one of the two predominant cell types in the alveolar area [67]. Promonocytic human histiocytic lymphoma cell line U937, depending on the initiators, might develop into either macrophages or monocyte or dendritic cells (antigen-presenting cells) [68]. Figure 7 shows the percentage of live cells in KETO and samples F0–F2 on both cell lines (A) U937 and (B) A549. The cytotoxicity was assessed in concentrations varying from 0.1 to 500 µg/mL. In terms of the A549 cells, all samples showed low cytotoxicity at a 50 and 5 µg/mL concentration, which was demonstrated by the % cell viability values being between 85 and 115%. Meanwhile, at 500 µg/mL, samples F0, F0.5, F1, and F2 had a % cell viability of 20%, 63%, 88%, and 60%, respectively. It was noticed that MAN enhanced the cell viability, and the lowest cytotoxicity was recorded in sample F1. On the other hand, all samples (including the raw KETO) showed high toxicity at a 500 µg/mL concentration with the U937 cell line; however, the cell viability was doubly enhanced in samples F1 and F2. At a concentration of 50 µg/mL, samples recorded a % cell viability between 63 and 83%, while at a concentration of 5 µg/mL, a high % cell viability was found (85–125%) with U937. These results aligned with previously published studies [69]. Nevertheless, our formulations exhibited very low cytotoxicity towards both cell lines, with live cell percentages between 85 and 125% at 5 µg/mL, which is the concentration used for the anti-inflammatory effect (Section 2.15).

### 2.15. Anti-Inflammatory Effect

**Figure 8:**
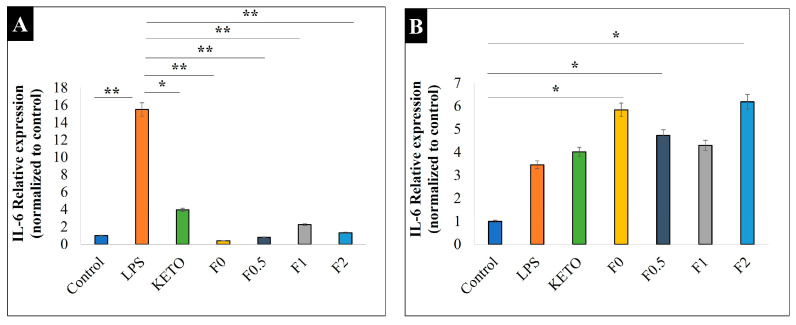
Relative expression of IL-6 on two cell lines: (**A**) U937 and (**B**) A549. Control is the untreated cell line, LPS is the treated cell line, KETO, F0, F0.5, F1, and F2 are treated with LPS. Results are expressed as mean ± SD (n = 3 independent measurements). Level of significance: (* *p* < 0.05), (** *p* < 0.01).

We determined IL-6 relative expressions on LPS-stimulated and -treated A549 and U937 cells to compare the anti-inflammatory effects of our samples. Here, LPS, a key activator of the proinflammatory response [70], was used to induce IL-6, a cytokine produced in acute and chronic pulmonary inflammations [71]. Both A549 and U937 cells can express IL-6 upon stimuli, such as LPS or cytokines [72,73]. In addition, U937 cell lines are widely used to model inflammation upon response to various compounds, as they contain higher amounts of inducible COX-2 enzyme compared to epithelial cells and show high reactivity to LPS [74,75]. Figure 8 illustrates the IL-6 relative expression of our samples on an LPS-treated U937 cell line (Figure 8A) and LPS-treated A549 (Figure 8B). All samples showed a significant anti-inflammatory effect and decreased IL-6 on the LPS-treated U937 cell line. Moreover, it was noted that the MAN in samples F0.5, F1, and F2 had no significant difference in anti-inflammatory effect compared to F0, indicating a promising combination. Here, we revealed that after LPS stimulation, A549 cells expressed significantly lower IL-6 compared to U937 cells (relative expression of 3.4 vs. 15.5, *p* < 0.01). Therefore, none of our treatments on LPS-stimulated A549 cells were able to decrease the IL-6 expression at the transcriptional level, which was in line with previously published results [69].

## 3. Discussion

The pulmonary route of administration for local delivery to target lung diseases offers many advantages over traditional routes. However, many pulmonary diseases need to be targeted with more than one pharmaceutical agent, making this a burden for patients who are required to inhale multiple drugs. In order to improve patient compliance, minimize the number of different dosing regimens, and gain better disease control, products comprising a combination of medications to be administered by a single inhaler have been created [76]. However, combining multiple drugs in one particulate system faces different challenges (i.e., high doses), and many attempts are being made to overcome these [77].

In this work, our goal was to create a novel combination consisting of mannitol and ketoprofen in a “nanoparticle-embedded coated-microparticle” system, in which mannitol served as the surface coating layer and ketoprofen nanoparticles were integrated into leucine. We successfully utilized distinctive particle engineering techniques to develop a combined DPI for targeting pulmonary inflammations that are linked with mucus overproduction. Ketoprofen, a poorly water-soluble NSAID, was first nanosized with PVA and SDS by ultraturrax and the wet-milling method, then co-spray dried with leucine and mannitol. Different ratios of mannitol were studied to evaluate its effect on the habits of ketoprofen nanoparticles and to confirm the compatibility of this combination.

Both in vitro and in silico deposition studies were carried out to make sure that the aerosol performance of ketoprofen was not altered after being combined with mannitol. Thanks to the nano-in-micro system, excellent in vitro aerodynamic performance was recorded, with ~71% FPF and ~2.5 µm MMAD. The in vitro aerosol performance results are considered promising and in line with other studies [78]. The highest lung deposition fractions in the in silico study were found in F0.5 and F1, which are correlated with in vitro results.

A significant enhancement in solubility in the developed formulations compared to raw ketoprofen was obtained. However, we believe that the solubility improvement was due to the nanosize of the ketoprofen, since no significant difference was found in F0 compared to mannitol-containing samples (F0.5–F2). The solubility results exhibited a better dissolution profile. Accordingly, a fast dissolution rate was achieved, in which 80% of the drug was released in 10 min due to the nanosize of the ketoprofen. In order to lower the chance of clearance mechanisms, it is well established in pulmonary administration that the administered drug needs to be rapidly dissolved and released upon lung deposition [38,58]. Moreover, all samples showed a significant anti-inflammatory effect and decreased IL-6 on the LPS-treated U937 cell line, with low toxicity indicating their safety. This reveals that mannitol did not amend the anti-inflammatory activity of ketoprofen.

The mannitol-containing samples decreased the viscosity of 10% mucin solution, while F1 showed the lowest viscosity. Because of the mannitol, the decreasing mucin viscosity can be attributed to increased hydration, and it can also lead to better mucus clearance. The relationship between the amounts of mannitol in our samples and the viscosity of sputum taken from individuals with COPD and CF should be further investigated. Also, a high permeation percentage (five-fold enhancement) was achieved in sample F1. Interestingly, the presence of mannitol in a high concentration (F2) showed a negative influence in terms of deposition, diffusion, and viscosity. This indicated that mannitol in high concentrations might be loaded less efficiently into the system, and thus, it should be carefully optimized. Consequently, we recommend this combination in a maximum mass ratio of 1:1 (F1).

Hence, our study showed that inhaled mannitol-coated ketoprofen as a nano-in-micro system can be a promising candidate for targeting pulmonary inflammations. This study is considered a fundamental base for further in vivo investigations. In addition, it can be performed for effectively combining various drugs to be delivered by inhalation.

## 4. Materials and Methods

### 4.1. Materials

KETO (TCI chemicals, Shanghai, China) was used as a drug model. Poly-vinyl-alcohol PVA (ISP Customer Service GmBH, Cologne, Germany) was used as a stabilizer. SDS (VWR chemicals, Leuven, Belgium) was employed as a surfactant. In spray-drying process, MAN (Molar Chemicals Ltd., Budapest, Hungary) was applied as a coating material, while LEU (AppliChem GmbH, Darmstadt, Germany) was exploited as a dispersity enhancer. Distilled water used in this study was obtained from Milli-Q, Millipore, Merck KGaA, Darmstadt, Germany.

### 4.2. Preparation of Ketoprofen-Containing Nanosuspension (KN)

**Figure 9:**
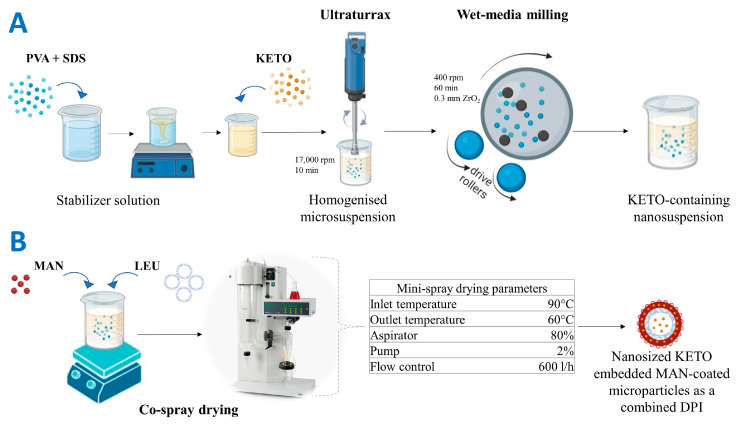
Preparation methods using particle engineering techniques: (**A**) preparation of the pre-dispersion (ketoprofen-containing nanosuspension), (**B**) preparation of nanosized ketoprofen-embedded mannitol-coated microparticles as combined dry powder for inhalation. KETO: ketoprofen, MAN: mannitol, LEU: leucine.

Ketoprofen-containing nanosuspension was prepared by wet-media milling process combined with a prior homogenization step. Wet-media milling is an attractive particle engineering approach which enables a simple scale-up in terms of industrial nanosuspension manufacturing [79]. The nanosuspension preparation process is illustrated in Figure 9A. Based on a preliminary study (Table S1), 1% PVA (*w*/*v*) combined with 0.1% SDS (*w*/*v*) was selected for the dispersant medium. PVA is a polymer used as a stabilizing agent in media milling. It reduces the cohesive forces between the milled particles and can enhance the lung deposition upon respiration [80,81]. SDS is a popular anionic surfactant used in the creation of core–shell nanoparticles, and it maintains the electrostatic stability during the milling process [33,82,83], so it was combined with the PVA in milling process.

First, 2 g of PVA and 0.2 g of SDS were dissolved in 200 mL of distilled water. Then, 2 g of KETO was suspended in 18 g of the stabilizer solution, and then homogenized at 17,000 rpm for 10 min using Ultraturrax (T-25, IKA-Werke, Breisgau, Germany). This step was employed for primarily suspending KETO in the dispersant medium [84]. After that, the coarse suspension (10% KETO content (*w*/*w*)) was milled with 20.00 g of zirconium dioxide (ZrO_2_) beads (0.3 mm), which was used as a milling media in a planetary ball mill (Retsch Planetary Next Ball Mill PM 100 MA, Retsch GmbH, Haan, Germany). The milling process was conducted at 400 rpm for 60 min in 50 mL milling chamber [85]. After milling, the nanosuspension was washed with the remaining stabilizer solution and filtered using 150 µm mesh size sieve to eliminate the bead particles. In the end, a KETO-containing nanosuspension (KN) with 1% (*w*/*v*) drug content was prepared.

#### Holding Time as Short-Term Stability of KN

To evaluate the stability of KN before co-spray drying process, a short-term stability test was carried out. KN was kept at room temperature (25 °C) and stored in refrigerator (+4 °C), and then it was evaluated in terms of PS, PDI, and ZP at definite time points using Malvern Zeta sizer Nano ZS (Malvern instrument, Malvern, UK).

### 4.3. Preparation of a Combined Dry Powder Inhaler (DPI)

Samples of dry powder for inhalation were prepared by a co-spray drying process (Mini Spray Dryer, Büchi B-191, Switzerland) with the parameters shown in Figure 9B. The parameters were optimized based on a preliminary study (Table S2). LEU was added in this step with a fixed mass ratio in all samples of 1:1 (LEU:KETO). LEU is a well-known excipient used in DPI formulation, since it decreases the interparticle forces and enhances the flowability, atomization, and dispersity [86]. LEU was used in our formulation as a surface-modifying agent by forming a rough encapsulating layer to improve the deposition in the lungs [87]. Moreover, many studies have confirmed that LEU increases the yield and stability of drugs and reduces the hygroscopicity of DPI [88,89,90]. LEU encapsulated KET particles by forming nano-in-micro composite. Mannitol is a low-hygroscopic sugar alcohol, which can be used in DPI formulation as a bulking agent, cryoprotectant, or coating agent, and its safety for pulmonary delivery was proven [88,91,92]. Furthermore, MAN is used as a mucolytic agent (add-on therapy for cystic fibrosis) [93]. In this study, MAN was using as a coating layer in which nanoembedded coated microparticles system was produced. MAN was added in different concentrations to evaluate its effect on the habit of KETO and to optimize the best concentration. Four samples with mass ratios of 0.5:1, 1:1, 2:1 (MAN:KETO), and with no mannitol were prepared and further investigated. The resultant spray-dried samples were named according to their MAN concentration: F0, F0.5, F1, and F2.

#### Yield and Drug Content of DPI

The mass ratio of dry powder collected after spray-drying to the initial solid compositions before drying was calculated to determine the percentage yield of each sample. For drug content calculation, a precisely scaled amount of the spray-dried samples was dissolved in 10 mL of distilled 50% methanol and stirred at 400 rpm by a magnetic stirrer (AREC. X heating magnetic stirrer, Velp Scientifica Srl, Italy) at room temperature for 24 h. Then, samples were filtered by filtration disks (0.45 µm pore size, Millex-HV syringe-driven filter unit, Millipore Corporation, Bedford, MA, USA) and quantified spectrophotometrically at λ 258 nm by UV (ATI-Unicam UV/VIS Spectrophotometer, Cambridge, UK). The percentage of ketoprofen content was determined as a ratio of calculated to theoretical drug content.

### 4.4. Characterization and Evaluation

#### 4.4.1. Particle Size, Particle Size Distribution, and Zeta Potential Characterization

Malvern Zeta sizer Nano ZS (Malvern instrument, UK) was used to characterize the particle size (PS), polydispersity index (PDI), and zeta potential (ZP) of the KN and DPI samples. KN was diluted 1:3 before analysis, and DPI samples were redispersed in distilled water. The refractive index was set to 1.592. Disposable cuvette cells (DTS1070) were used. All samples were measured at 25 °C in three parallel runs, and the average was evaluated.

#### 4.4.2. Solubility

The solubility study was conducted by adding excess amounts of KETO and dry powder samples in 5 mL of distilled water. Samples were stirred at 400 rpm for 24 h at room temperature using a magnetic stirrer (AREC. X heating magnetic stirrer, Velp Scientifica Srl, Italy), and then filtered by filtration disks (0.45 μm, Millex-HV syringe-driven filter unit, Millipore Corporation, Bedford, MA, USA). KETO concentration in samples was analyzed by UV/VIS spectrophotometer (ATI-Unicam UV/VIS Spectrophotometer, Cambridge, UK) at λ 258 nm. All samples were measured in triplicate.

#### 4.4.3. Morphology

Morphology of the spray-dried powder samples (F0–F2) was detected using scanning electron microscopy (SEM) (Hitachi S4700, Hitachi Scientific Ltd., Tokyo, Japan) at 10 kV. A sputter coater (Bio-Rad SC 502, VG Microtech, Uckfield, UK) was used to coat the samples with gold-palladium with 2.0 kV electric potential, at 10 mA amperage and for 10 min. Air pressure was adjusted to 1.3–13.0 mPa. According to SEM results, diameter size of the final product particles was investigated using image analyzer software (ImageJ) (https://imagej.net/ij/index.html, accessed on 15 March 2023). Moreover, to define the habit of materials used in this system without KETO, blanks (PVA_SDS, PVA_SDS_LEU, PVA_SDS_MAN, and PVA_SDS_LEU_MAN) were spray-dried with the same parameters mentioned earlier and then analyzed by SEM.

#### 4.4.4. Contact Angle, Surface Energy, and Cohesion Work

Interparticle interactions of the dry powder samples and KETO were measured in terms of contact angle (CA), surface energy (Υ), polarity (Pol), and cohesion work (Wc). One-ton hydraulic press (Specac Inc. in Waltham, MA, USA) was used to press about 0.10 g of the dry powder samples into pastilles. Out of each sample, six pastilles were produced. Polar (10 µL of distilled water) and non-polar (2.0 µL of diiodomethane) solvents were employed to drip onto the surface of pastilles using a vertical electronic syringe. Each sample was subjected to three parallel measurements. Using a Dataphysics OCA 20 device (Dataphysics Instrument GmbH, Filderstadt, Germany), contact angle was detected from the two applied liquids in a range of 1 to 30 s. The surface energy (Υ), which is composed of a polar part (Υp) and a dispersive part (Υd), was determined using SCA20 software (version 5.0.41 build 5041, Data Physics Instruments, GmbH, Filderstadt, Germany) [43]. Polarity (Pol) was calculated by Equation (1), while cohesion work (Wc) was calculated by Equation (2).

Pol = (Υp)/(Υ) × 100(1)

Wc = (Υ) × 2(2)

#### 4.4.5. Thermal Analysis

##### DSC

Differential scanning colorimetry (DSC) assessment was carried out by a Mettler Toledo DSC 821e thermal analysis system with the help of STARe thermal analysis program version 9.3 (Mettler Inc., Schwerzenbach, Switzerland). A mass of 2–3 mg of each sample and raw materials was scaled into DSC aluminum pans, which were hermetically sealed, and lid pierced. An empty pan was used as a reference. The measurement was conducted between the temperature range of 25 °C and 300 °C. The flow rate of the carrier gas (Argon) was 10 L/h, while the heating rate was 10 °C/min.

##### TGA

A thermal analysis system (TGA) was used to determine the residual water content in our samples based on loss of drying concept [91]. Mettler–Toledo TGA/DSC1 (Mettler–Toledo GmbH, Greifensee, Switzerland) instrument connected with a quadrupole mass spectrometer (MS, Pfeiffer Vacuum GmbH, Asslar, Germany, model ThermostarTM GSD 320) was used in this measurement. STARe thermal analysis program V9.3 (Mettler Inc., Schwerzenbach, Switzerland) was used to evaluate the data. About 2–3 mg of samples was measured between 25 and 300 °C with a heating rate of 10 °C/min. Contact between the MS and the TG was maintained at 120 °C and performed with a silica capillary. The investigation was carried out in an atmosphere of argon with a constant gas flow (10 mL/min).

#### 4.4.6. Structural Analysis

Spray-dried samples and raw materials were characterized using a BRUKER D8 Advance X-ray powder diffractometer (Bruker AXS GmbH, Karlsruhe, Germany) with a radiation source of Cu K λ1 (λ = 1.5406 Å) and a VÅNTEC-1 detector. Dry powder samples were scanned with Cu target and Ni filter at a voltage of 40 kV and a current of 40 mA, throughout 3° to 40° 2θ angular phase, at 0.1 s step time and 0.01° step size. Crystallinity index (%Xc) was used to calculate the crystallinity degree of our samples compared to the raw materials (Equation (3)), where A is the area under the curve and pure KETO was considered a 100% crystalline.

%Xc = A_crystalline_/(A_crystalline_ + A_amorphous_) × 100% (3)

#### 4.4.7. Density and Flowability

Dry powder samples were characterized by tapped and bulk densities. An Engelsmann Stampfvolumeter (Ludwigshafen, Germany) instrument was used for this assessment. Samples were accurately scaled and filled in a graduated cylinder (10 cm^3^) to measure the bulk density (ρb), which it was calculated by dividing the powder mass over the untapped volume (m/v_0_). The tapped (ρt) density was measured after 1250 times tapping and calculated by dividing the mass over the tapped volume (m/v_f_) [94,95]. Furthermore, Carr’s index (CI) and Hausner ratio (HR) were used to study the flowability characteristics of our samples (Equations (4) and (5)). All samples were measured in triplicate.

CI = (ρt − ρb)/ρt × 100(4)

HR = ρt/ρb(5)

#### 4.4.8. Characterization of Aerosol Performance

##### In Vitro Aerodynamic Characterization

Andersen Cascade Impactor (ACI) (Copley Scientific Ltd., Nottingham, UK) was used to study the aerosolization characteristics of the dry powder samples [96,97]. The inhalation flow rate was set to 60 L/min, which was generated by a vacuum pump (High-capacity Pump Model HCP5, Critical Flow Controller Model TPK, Copley Scientific Ltd., Nottingham, UK), and a mass flow meter (Flow Meter Model DFM 2000, Copley Scientific Ltd., Nottingham, UK) was used to confirm the actual flow rate during the inhalation. Dry powder samples containing 5 mg of KETO, which is one-tenth of its oral dose, were filled in two size 3 Ezeeflo™ hydroxypropyl methylcellulose capsules (ACG-Associated Capsules Pvt. Ltd., Mumbai, India) [60]. Capsules were loaded into a Breezhaler single-dose device and actuated before inhalation, while the inhalation time was 4 s. All the stage plates were soaked in a mixture of Span 85 and cyclohexane (1:99 *v*/*v*%) to mimic the adhesive properties in the lung tissues, and then, they were left to dry. After applying the inhalation, all parts of the ACI were washed using a solution of methanol and water (1:1 *v*/*v*%) in order to collect and dissolve the deposited mass of KETO. The concentration of KETO deposited in each stage was measured using UV/VIS spectrophotometry (ATI-UNICAM UV/VIS Spectrophotometer, Cambridge, UK) at λ = 258 nm. Inhalytix™ (Copley Scientific Ltd., Nottingham, UK) software (Available online: https://www.copleyscientific.com/inhaler-testing/apsd-data-analysis-software/inhalytix/, accessed on 15 January 2023) was used to evaluate the in vitro aerodynamic properties of our samples. Different values were considered to evaluate the aerodynamic profile of our samples. MMAD was used to express the real size of the particles during inhalation, FPF expressed the percentage of the mass of drug particles with a size less than 5 µm and the amount of drug leaving the device and reaching the impactor, and EF, which is a percentage of the amount of drug leaving the device and reaching the impactor divided by the initial amount of loaded KETO.

##### In Silico Characterization

The most recent version of the Stochastic Lung Model (SLM) was applied for determining the amount of drug deposited in various anatomical regions of the airways, which are represented as regional deposition fractions [98]. Numerical modeling is a compliant, non-invasive, and reproducible method that is advantageous compared to other systematic scintigraphic studies which need a large population and have many barriers (i.e., technical and ethical barriers) [99]. Recently, numerical models turned out to be an effective tool for quantifying the distributions of various medications in the total, local, and regional respiratory tract depositions. In this study, a validated airway deposition model was used to calculate numerically the deposition fractions in the lungs and upper airways (extra-thoracic region). Deposition fraction can be defined as the proportion of drug mass deposited in a specific area of the airways to drug mass loaded in the capsule. The lung deposition fraction is the sum of bronchial and acinar deposition fractions. The exhaled fraction was calculated by subtracting the total of the fractions deposited in the lungs and upper airways and the fraction that remained in the device from the ratio of the metered fraction (100%). A Monte Carlo approach for choosing particle routes and realistic observed geometric parameters allows the computer model to reproduce the underlying morphological stochasticity of the airways. Typically, 105 particles are monitored from inhalation until expiration, where they deposit in different lung regions or are exhaled. Particle characteristics and inhalation parameters are the main inputs of this model. The results of in vitro aerodynamic assessment that we gained from ACI were utilized for this study. Inhalation parameters used for this model were as follows: peak inhalation flow: 69.5 L/min, inhaled volume: 1.7 L, and inhalation time: 2.04 s. Those parameters were matched to patients with chronic obstructive pulmonary disease [100]. Moreover, in order to assess the impact of breath hold (BH) duration time on the deposition manner, 5 s and 10 s BH times were employed. In our prior work, the numerical deposition model was verified specifically for the circumstance of aerosolized medicines [101].

#### 4.4.9. In Vitro Release Study

A modified paddle method (Hanson SR8 Plus, Teledyne Hanson Research, Chatsworth, CA, USA) from European Pharmacopeia was applied to study the release of KETO [102]. A 100 mL vessels’ volume instead of 1000 mL was used with smaller paddle size. A simulated lung fluid (SLF), which was composed of NaCl, NaHCO_3_, CaCl_2_, NaH_2_PO_4_, H_2_SO_4_, and glycine (pH = 7.4), was prepared [103]. Vessels were filled with 50 mL of SLF, and temperature was set to 37 °C. These parameters were designed based on the human airways’ circumstances [104,105]. The raw drug and samples equivalent to 5 mg of KETO (based on drug content analysis) were dispersed in the medium. We decided the pulmonary dose of KETO as 10% of its oral dose [60]. The rotation of paddles was adjusted to 50 rpm, and the duration of the study was 120 min. A total of 2 mL of each sample was withdrawn and replenished after 5, 10, 15, 30, 60, and 120 min. Samples were filtered (filtration disks with 0.45 µm pore size, Millex-HV syringe-driven filter unit, Millipore Corporation, Bedford, MA, USA), and then quantified spectrophotometrically at λ 258 nm (UV/VIS ATI-UNICAM UV/VIS Spectrophotometer, Cambridge, UK). Measurements were carried out in triplicate.

#### 4.4.10. In Vitro Diffusion Study

A modified horizontal diffusion cell was used to assess the in vitro permeability of KETO and dry powder samples from the lung fluid to the epithelial cells of the lung. The cells used in this study were 3D-printed unique structures developed by the research group [106]. To model the lung fluid, 9 mL of SLF (pH = 7.4) was used as a donor phase, while 9 mL of phosphate-buffered solution (pH = 7.4) was used as an acceptor phase to model the epithelial cell. A cellulose membrane (RC 55 WhatmanTM GE Healthcare Life Sciences, Buckinghamshire, UK) with 0.5 μm pore size and 0.75 μm thickness was soaked in isopropyl myristate for 30 min and then placed between the two phases. To ensure the homogeneous distribution of the samples and model the lung circumstances, a continuous stirring and a maintained temperature were run throughout the experiment. The temperature was set to 37 °C with the help of a water-circulator thermostat. The magnetic stirrer (CS-Smartlab Devices Ltd., Kozarmisleny, Hungary) was adjusted at 150 rpm, while the total diffusion surface area was 0.785 cm^2^. Dry powder samples equivalent to 5 mg of KETO and 5 mg of raw KETO were dispersed in the donor phase, and the diffused amount was real-time quantified with a probe immersed in the acceptor phase (FDP-7UV200-VAR, Avantes, Apeldoorn, The Netherlands) at 258 nm by a UV/VIS spectrophotometer (Avaspec-ULS2048-USB2, Avantes, Apeldoorn, The Netherlands). The duration of the measurement was 60 min. Measurements were conducted in triplicates. The flux (J) was calculated from the KETO amount diffused through the membrane (m) divided by the surface area of the membrane (A) and the total duration of the experiment (t) (µg/cm^2^/h), as seen in Equation (6). Moreover, the relative permeation at 60 min (RP60) was calculated as a ratio of diffused amount from our samples compared to the control (raw KETO).

J = m/(A × t)(6)

#### 4.4.11. Effect on Mucin Viscosity

Mucin is the major component in the mucus of respiratory system [107]. Assessing mucin viscosity is essential for the diagnosis, management, and treatment of various respiratory conditions. Therefore, mucin was prepared in different concentrations, and then, spray-dried samples were added to evaluate their effect on the mucin viscosity. Mucin from porcine stomach type II (Sigma-Aldrich, Merck. Ltd., Saint Louis, MO, USA) was used. IKA viscometer instrument (IKA, Rotavisk, IKA-Werke GmbH & Co. KG, Staufen, Germany) was employed to check the viscosity of mucin with and without the addition of our samples. A thermostatic circulator (IKA-HRC II control + PT 100.30 Temperature sensor) was connected to the equipment to ensure a constant temperature during the measurement. Samples containing 5 mg of KETO were added to the mucin solution and stirred for 30 min. Spindle number 11 (SP-11) was used, while parameters of 120 rpm and 30 s were set. The experiment was conducted at 37 °C in three parallel runs, and the average (±SD) was evaluated.

#### 4.4.12. Cytotoxicity

To determine the effect of our samples, KETO, and the excipients on cell viability, the MTT staining method, described earlier by Mosmann (1983) [108], was performed on two types of cell line. A549 human airway epithelial cells and U937 promonocyte cells (ATCC, Manassas, VA, USA) were transferred to a 96-well plate at a density of 4 × 10^4^ cells/well in 100 µL of minimal essential medium (MEM) with Earle’s salts or RPMI supplemented with 10% heat-inactivated fetal bovine serum (FBS), 2 mmol/L-glutamine, 1× non-essential amino acids, 4 mM HEPES, and 25 µL/mL gentamycin. Prior to the study, the adherent cells were cultured for 24 h at 37 °C, with 5% CO_2_ in the above described medium. The next day, the medium was removed, and then the cells were treated with increasing concentrations of our samples, which were two-fold diluted in MEM or RPMI. The starting concentration of the samples and KETO was 500 µg/mL, and the concentration range was between 500 and 0.97 µg/mL. Culture media alone served as negative control and untreated cells were used as positive control. To all conditions, internal triplicates were included. The culture plates were incubated at 37 °C for 24 h. After the incubation period, 20 µL of MTT (thiazolyl blue tetrazolium bromide, Sigma) solution (from a stock solution of 5 mg/mL) was added to each well, and plates were incubated again for 4 h at 37 °C. At the end of the incubation period, 100 µL of SDS (Sigma) solution (10% in 0.01 M HCI) was added to each well. After a further 24 h overnight incubation period at 37 °C with 5% CO_2_, the cell viability was determined by measuring the optical density (OD) at 570/650 nm with EZ READ 400 ELISA reader (Biochrom, Cambridge, UK). Inhibition of cell growth was expressed in IC50, which was calculated based on Equation (7) and plotted against the logarithm of concentrations. Cell viability was expressed in % control cells and measured according to Equation (8). GraphPad Prism 8.0.1. software (GraphPad Software Inc., San Diego, CA, USA) was used for statistical analysis and visualization.

100 − ((OD_sample_ − OD_medium control_)/(OD_cell control_ − OD_medium control_)) × 100 (7)

%Control = (Absorbance of sample)/(Absorbance of control) × 100(8)

#### 4.4.13. Anti-Inflammatory Effect

A549 cells were propagated in Eagle MEM (Sigma, St. Louis, MO, USA) media, and U937 cells were grown in RPMI (Thermo Scientific, Waltham, MA, USA) media, supplemented with 25 μg/mL gentamycin, 10% foetal calf serum, 0.5% *w*/*v* glucose, 0.3 mg/mL l-glutamine, and 4 nm HEPES. The cells were transferred to 6-well plates at a density of 1 × 10^6^ cells/well. Each sample was used in triplicates. The cells were treated with 5 μg/mL LPS (Thermo Scientific, Waltham, MA, USA) and with one of the following: KETO (5 μg/mL) or F0, F0,5, F1, or F2 (equivalent to 5 μg/mL of KETO). LPS-treated cells served as positive control, while negative control cells were left untreated. All plates were incubated for 48 h at 37 °C, 5% CO_2_ before use.

##### mRNA Extraction and cDNA Synthesis

Total RNA was extracted from treated and control cells after a 48 h incubation period using TRI reagent (Sigma-Aldrich, Saint Louis, MO, USA) according to the manufacturer’s protocol. Total RNA concentrations and purity were measured using a NanoDrop spectrophotometer (Thermo Scientific, Waltham, MA, USA). First-strand cDNA was synthesized using Maxima First Strand cDNA Synthesis Kit (Thermo Fisher Scientific Inc., Waltham, MA, USA) and 20 pM random hexamer primer according to the manufacturer’s protocol (Thermo Fisher Scientific Inc., Waltham, MA, USA).

##### qPCR Validation of IL-6

qPCR was performed in a Bio-Rad CFX96 real-time system with SsoFast™ Eva-Green^®^qPCR Supermix (Bio-Rad, Hercules, CA, USA) master mix, using the following human specific primer pairs: IL-6 sense: 5′-CAGCTATGAACTCCTTCTCCAC-3′ and Il-6 antisense 5′-GCGGCTACATCTTTGGAATCT-3′, Actb sense 5′-TTCTACAATGAGCTGCGTGTGGCT-3′ and Actb antisense 5′-TAGCACAGCCTGGATAGCAACGTA-3′. Cycle threshold (Ct) values were calculated for β-actin and Il-6, and the relative gene expression levels were determined by the 2^−ΔΔCt^ method. The relative expression level was indicated as 2^−ΔΔCt^, where ΔΔCt = ΔCt for the experimental sample and −ΔCt for the control sample. Statistical analysis of data was performed with GraphPad Prism 8.0.1. software, using one-way ANOVA. *p* value < 0.05 was considered statistically significant.

## 5. Conclusions

In this study, a novel inhalable nanoembedded coated microparticle system using particle engineering technologies was produced. Common pulmonary inflammations can be lethal, particularly if they are associated with unusual mucus accumulation. This work revealed that inhalation of mannitol and ketoprofen together from a single inhaler is feasible and promising for targeting pulmonary inflammations. This approach is fundamental and supports further in vivo studies.

## Figures and Tables

**Table 1:** Sample notations, description, yield of spray-dried powder, and drug content.

Sample Name	Sample Description	Yield (%)	Drug Content (%)
F0	KETO1_LEU1_MAN0	52.95 ± 3.54	57.47 ± 1.44
F0.5	KETO1_LEU1_MAN0.5	54.86 ± 6.12	84.12 ± 2.91
F1	KETO1_LEU1_MAN1	57.29 ± 1.98	84.87 ± 7.13
F2	KETO1_LEU1_MAN2	58.68 ± 9.73	84.72 ± 3.65
KETO	Ketoprofen_raw	-	-

LEU: L-leucine, MAN: mannitol. Results are expressed as mean ± SD (n = 3 independent measurements).

**Table 2:** Particle analysis: particle size (PS), polydisperse index (PDI), and zeta potential (ZP).

Sample	PS (nm)	PDI	ZP (mV)
F0	204.9 ± 3.07	0.336 ± 0.003	−8.88 ± 0.27
F0.5	222.5 ± 4.11	0.127 ± 0.021	−12.3 ± 0.43
F1	240.7 ± 6.32	0.064 ± 0.008	−7.44 ± 0.18
F2	251.4 ± 2.84	0.156 ± 0.039	−11.9 ± 0.33

Results are expressed as mean ± SD (n = 3 independent measurements).

**Table 3:** Solubility of spray-dried samples and KETO.

Sample Name	Solubility * (mg/mL)
F0	13.93 ± 0.88
F0.5	17.77 ± 1.05
F1	15.04 ± 0.34
F2	17.95 ± 1.71
KETO	0.42 ± 0.13

* Results are expressed as mean ± SD (n = 3 independent measurements).

**Table 4:** Density, flowability, and in vitro aerodynamic characteristics of spray-dried samples.

Sample Name	F0	F0.5	F1	F2
Bulk Density (g/cm^3^)	0.124 ± 0.012	0.123 ± 0.003	0.120 ± 0.031	0.139 ± 0.024
Tapped Density (g/cm^3^)	0.180 ± 0.002	0.192 ± 0.011	0.201 ± 0.009	0.228 ± 0.052
Carr’s Index	31.03	35.14	40.01	39.01
Hausner Ratio	1.450	1.542	1.670	1.64
MMAD (µm)	2.40 ± 0.17	2.80 ± 0.06	4.51 ± 0.41	4.90 ± 0.16
FPF (%)	56.16 ± 2.51	71.02 ± 1.19	64.32 ± 1.34	32.21 ± 3.67
EF (%)	97.06 ± 3.22	96.60 ± 1.65	94.82 ± 2.79	95.70 ± 2.89

MMAD: mass median aerodynamic diameter, FPF: fine particle fraction, EF: emitted fraction. Results are expressed as mean ± SD (n = 3 independent measurements).

**Table 5:** In vitro permeation results of raw KETO and spray-dried samples in simulated lung media, pH = 7.4. Flux (J), permeability coefficient (Kp), and relative permeability at 60 min (RP60).

Sample	J * (µg/cm^2^/h)	RP60	Kp (cm/h)
KETO	24.79 ± 5.29	1.000	-
F0	98.24 ± 11.34	3.96	0.896
F0.5	74.18 ± 18.63	2.99	0.470
F1	121.97 ± 23.12	4.92	0.877
F2	30.04 ± 16.58	1.21	0.336

* Results are expressed as average ± SD (n = 3).

**Table 6:** Viscosity results of 10% mucin solution before and after adding the spray-dried samples.

Sample	Viscosity (Pa·s)
Mucin 10%	0.035 ± 5.44
F0	0.033 ± 1.98
F0.5	0.031 ± 2.12
F1	0.025 ± 1.37
F2	0.030 ± 4.19

Results are expressed as average ± SD (n = 3).

## Data Availability

Data is contained within the article and Supplementary Material.

## Supplementary Materials

The following supporting information can be downloaded at https://www.mdpi.com/article/10.3390/ph17010075/s1: Table S1: A preliminary study of ketoprofen-containing nanosuspension using different stabilizers and concentrations; Table S2: Spray dryer parameters’ impact on yield, particle size, and polydispersity index.
