# Supplementary material for: A Novel Combined Dry Powder Inhaler Comprising Nanosized Ketoprofen-Embedded Mannitol-Coated Microparticles for Pulmonary Inflammations: Development, In Vitro–In Silico Characterization, and Cell Line Evaluation
Source: Pharmaceuticals (Basel). 2024 Jan 7;17(1):75. doi: 10.3390/ph17010075 (PMC10818896; doi:10.3390/ph17010075)
Supplement: Supplementary file 1 [file pharmaceuticals-17-00075-s001.zip › pharmaceuticals-2807311-supplementary.pdf]

Table S1. A preliminary study of ketoprofen-containing nanosuspension using different stabilizers and concentrations

**Stabilizer/Conc. (w/v)***	**PDI**	PS **(nm)**	ZP (mV)
**HPMC**			
**0.25**	0.454 ± 0.11	2570.0 ± 42.6	-5.5 ± 1.34
**0.5**	0.739 ± 0.02	764.3 ± 2.79	-8.1 ± 4.24
**1**	0.319 ± 0.03	453.0 ± 4.15	-6.2 ± 2.87
**PVA**			
**1**	0.082 ± 0.01	238.3 ± 1.37	-9.97 ± 5.31
**2.5**	0.227 ± 0.04	316.4 ± 1.91	-11.6 ± 2.75
**5**	0.205 ± 0.02	431.4 ± 3.16	-4.22 ± 0.23
**Poloxamer 188**			
**0.2**	0.428 ± 0.14	583.2 ± 12.55	-13.1 ± 1.18
**0.5**	0.213 ± 0.05	3112.1 ± 36.44	-25.2 ± 3.17
**1**	0.321 ± 0.16	415.7 ± 13.06	-17.5 ± 2.94

*All stabilizers were combined with 0.1% SDS

Table S2. Spray dryer parameters impact on yield, particle size and polydispersity index

**Pump (%)***	**Temp. (C°)***	**Yield %**	**PS (nm)**	**PDI**
**10%**	**90**	45.58	610.2 ± 7.57	0.521 ± 0.117
**10%**	**70**	41.67	391.7 ± 3.15	0.838 ± 0.445
**10%**	**50**	46.98	415.4 ± 8.67	0.412 ± 0.717
**5%**	**90**	59.34	784.6 ± 4.38	0.217 ± 0.234
**5%**	**70**	48.12	303.1 ± 2.51	0.567 ± 0.342
**5%**	**50**	51.34	481.5 ± 9.26	0.193 ± 0.095
**2%**	**90**	52.95	204.9 ± 3.07	0.336 ± 0.003
**2%**	**70**	49.05	290.5 ± 3.07	0.269 ± 0.105
**2%**	**50**	55.93	354.8 ± 5.36	0.513 ± 0.111

*Feed concentration was fixed (1:1 ketoprofen:leucine)
